# Pain Acceptance in Adolescent Chronic Pain: Do Body Mindsets Play a Role?

**DOI:** 10.1097/AJP.0000000000001307

**Published:** 2025-07-01

**Authors:** Emily J. Dowling, Laura E. Simons, Alia J. Crum, Joshua Pate, Joseph Chilcot, Helen C. Laycock, Whitney Scott, Lauren C. Heathcote

**Affiliations:** * Department of Psychology, Health Psychology Section, Institute of Psychiatry, Psychology, and Neuroscience, King’s College London; ∥ Department of Paediatric Anaesthesia and Pain Medicine, Great Ormond Street Hospital, London, United Kingdom; † Department of Anesthesiology, Perioperative, and Pain Medicine, Stanford University School of Medicine; ‡ Department of Psychology, Stanford University, Stanford, CA; § Graduate School of Health, University of Technology Sydney, Sydney, NSW; ¶ INPUT Pain Unit, Guy’s and St Thomas’ NHS Foundation Trust

**Keywords:** mindset, body mindsets, paediatrics, adolescents, chronic pain, pain acceptance

## Abstract

**Objective:**

Pain acceptance predicts better quality of life, physical functioning, and treatment outcomes in youth with chronic pain. However, we know little about the factors that promote pain acceptance in youth. This study investigated body mindsets and their associations with facets of pain acceptance, specifically pain willingness and activity engagement, in adolescents with chronic pain.

**Methods:**

The sample comprised 102 adolescents with chronic musculoskeletal pain (aged: 8 to 17; 72.3% female, 49.5% Caucasian/White) attending a tertiary pain clinic. Hierarchical linear regression analyses examined associations of body mindsets with pain acceptance, controlling for demographic factors, pain and mental health symptoms, and basic functioning.

**Results:**

There was significant variation in the mindsets that adolescents with chronic pain held about their bodies—some endorsed the mindset that their Body is an Adversary, others endorsed the mindsets that their Body is Responsive or Body is Capable. Hierarchical linear regression analyses indicated that endorsing the mindset that their Body is an Adversary was associated with lower willingness to experience pain, while endorsing the mindset that their Body is Capable was associated with greater engagement in valued activities despite pain, even after accounting for demographic factors, pain characteristics, and basic functioning. Together, all 3 mindsets explained 6.6% to 26.8% unique variance in pain acceptance.

**Discussion:**

Body mindsets are significantly associated with pain acceptance in youth with chronic pain, even after controlling for pain characteristics and basic functioning. Experimental research should investigate whether body mindsets are modifiable in this population and whether they could represent interventional targets fostering pain acceptance.

Chronic pain affects 20% of children and adolescents, making early intervention crucial to prevent the transition of long-term pain in adulthood and reduce societal and health care burden.^1^ Pain acceptance can facilitate functional recovery from chronic pain by supporting individuals to shift away from unworkable efforts to control pain and towards prioritizing valued life goals while experiencing pain.^2^ Pain acceptance comprises 2 core facets: pain willingness, defined as responding to pain experiences without attempts at control or avoidance, and activity engagement, defined as engaging in valued life activities despite pain.^3^ Both are important for pain outcomes in adults^4,5^ and young people with chronic pain.^6,7^ Behavioral interventions targeting pain acceptance may be especially effective for adolescents, whose pain responses are less entrenched than adults and whose brain plasticity may enhance flexible thinking about pain.^8^

Novel behavioral interventions targeting pain acceptance show promise for improving pain outcomes in adolescents with chronic pain;^9,10^ identifying factors that foster pain acceptance could enhance these interventions.^11^ The Fear-Avoidance Model (FAM)^12^ and the psychological flexibility model^13^ propose risk and resilience factors that hamper or foster pain acceptance. The FAM suggests that individuals who interpret pain as threatening are more likely to experience pain-related fear and subsequent avoidance behaviors, which can perpetuate disability and distress. Within this model, low pain acceptance may be seen as a consequence of heightened threat appraisals and fear responses, reinforcing a cycle of avoidance and functional impairment.^14^ While positive and negative affect moderate pain acceptance in the FAM, they may be poor targets due to their transient and fluctuating nature.^15^ Similarly, personality traits—also indicated as moderators, are also unpromising targets as they are trait-like factors that are relatively unchangeable.^16,17^ In contrast, the psychological flexibility model emphasizes present-moment awareness, openness to pain-related experiences rather than avoidance, and values-based action. Within this, pain acceptance supports greater functioning and well-being even in the presence of pain. The psychological flexibility model argues that pain acceptance is not dependent on the specific content of pain-related thoughts and feelings and instead focuses on the function of behaviors in response to these experience.^18^ Nonetheless, it is plausible that specific pain- or body-related thoughts may enable greater acceptance to a degree.

A promising regulatory target that sits between state and trait-like factors are mindsets. Mindsets are core beliefs about a given domain that have downstream effects on attention, affect, motivation, and physiology, thereby influencing overall health.^19–21^ Mindsets about the body—termed “body mindsets”—are particularly relevant for people living with long-term conditions,^22,23^ including pain. Body mindsets are core assumptions about what the body is, how it functions, and what it represents. Although body mindsets share similarities with concepts like body image, they encompass a broader range of core beliefs, particularly regarding how the body functions rather than solely how it appears. These mindsets reflect personal perceptions of the body’s abilities or performance, making them distinct from objective physical characteristics. For instance, someone living with persistent and debilitating pain may understandably perceive their body as an “Adversary”—at fault and to blame for the pain they feel. Yet, someone else living with ongoing pain may still perceive their body as “Capable” of handling and dealing with pain. Similarly, experiencing recovery and resilience despite pain might reinforce a belief that their body is highly “Responsive.” In this sense, mindsets are not strictly true or false—they are interpretive lenses that shape attention, emotions, and behaviors, and they can be more or less useful in a given context.

In previous research, childhood cancer survivors who more strongly endorsed the mindset that their Body is an Adversary reported worse pain and greater bodily threat monitoring behaviors, while those who more strongly endorsed the mindset that their Body is Responsive engaged in less bodily threat monitoring behaviors.^22^ Mindsets have been shown to be amenable through brief, targeted digital interventions, leading to improved health-related motivation and well-being. For example, an intervention promoting helpful body mindsets in adults with painful knee osteoarthritis significantly improved movement-related fear, self-efficacy, and physical activity levels.^24^ Thus, mindsets show potential as adjuncts to chronic pain therapies.^25,26^

This study aimed to provide a preliminary examination of body mindsets and their cross-sectional association with facets of pain acceptance—activity engagement and pain willingness—in adolescents attending a tertiary pain clinic. We investigated 3 body mindsets: the more unhelpful mindset that the Body is an Adversary, and more helpful mindsets that the Body is Capable and the Body is Responsive. We hypothesized that the Body is an Adversary mindset would be associated with lower activity engagement and less pain willingness, while more helpful body mindsets would be associated with greater activity engagement and pain willingness. Lastly, we hypothesized that body mindsets would contribute significant variance in hierarchical linear regression analyses in pain acceptance beyond demographics, pain characteristics, and basic functioning.

## METHODS

### Participants

Eligible participants were aged 8 to 17 years old and seeking care at the Stanford Children’s Health Pediatric Pain Management Clinic. Participants were ineligible if they had significant cognitive impairment (self-reported or clinician-reported) or could not read or communicate in English. In total, 182 youth underwent an initial clinic evaluation and 127 of these youth (70%) provided consent and completed the study measures. Twenty-five (20%) of these youth did not complete the Body Mindset Inventory-Child Version (BMI-C), and thus data from 102 adolescents were analysed for this study. Adolescents who did not complete the BMI-C (M=23.33, SD=4.09) had significantly greater pain self-efficacy than adolescents who did not complete the measure (M=20.10, SD=5.80). No other significant differences were seen across other measures (see Table S1, Supplemental Digital Content 1, http://links.lww.com/CJP/B212).

### Procedure

The study received ethical approval from the Stanford Medicine Institutional Review Board (IRB-46499). Before initial evaluation, patients completed various online measures administrated through the Pediatric Collaborative Health Outcomes Information Registry (Peds-CHOIR).^27^ Peds-CHOIR is an open-source clinical database used as a health outcomes tracker and captures data regarding demographics, pain, and psychosocial functioning. During initial evaluation meetings, youth are assessed by a physiotherapist, a pain physician, and a pain psychologist who provide a diagnosis, recommendations, and a combined multidisciplinary pain management plan. Following these evaluations, a research coordinator introduced the study to adolescents and their families. Interested participants completed an online screening form through REDCap,^28^ and if eligible, they provided consent and enrolled onto the study. Participants completed study-specific self-report measures either on a tablet in the clinic waiting area or through a secure REDCap link. Surveys took up to 30 minutes, and participants received a $10 Amazon gift voucher and were entered into a raffle for the chance to win a $200 gift card. Data regarding demographics, pain, and mood were additionally extracted from Peds-CHOIR for the purpose of the study. Data from the current study sample has been used previously to conduct an initial validation of the Concept of Pain Inventory (COPI);^29^ data on body mindsets were not reported in the previous study. Measures capturing body mindsets and pain acceptance were central to the research questions in this study; however, additional pain-related risk and resilience factors that were collected as part of the overall cohort study were also included in the current research as additional exploratory measures to guide future studies.

### Measures

#### Demographics and Pain Characteristics

Data regarding age, sex assigned at birth, race, and pain characteristics were obtained from the Peds-CHOIR registry. Adolescents reported all the locations in which they had chronic pain. Pain location data was summarized into a binary variable; the localized pain group included adolescents with headache, abdomen, and localized musculoskeletal pain, while the diffuse pain group included adolescents with diffuse musculoskeletal and widespread pain. Pain frequency was measured on a 6-point Likert scale (0=never in the last month, 5=daily). Average pain intensity was measured using the Numeric Rating Scale (NRS-11) from 0 (no pain) to 10 (worst possible pain).^30^ Parents and adolescents reported the date the adolescent’s pain problem started, with the option to estimate or use the first day of the month for when they remembered the problem starting.

#### Body Mindsets

The Body Mindset Inventory-Child Version (BMI-C) comprises 8 items assessing 3 mindsets: the Body is Capable (3-items; α=0.73; eg, “My body can handle pain”), the Body is an Adversary (3-items; α=0.67; eg, “Having pain means that my body is letting me down”), the Body is Responsive (2-items; α=0.80; eg, “My body can heal itself”). The BMI-C can capture body mindsets in the context of any illness or health experience—for example, in a previous study conducted in children and young adults, the BMI-C items were oriented around the context of cancer.^22^ Similarly, the adult version has been used in a sample of individuals with chronic pain.^23^ In the current study, items were oriented around the context of pain, marking the first time the measure has been applied to children and adolescents with chronic pain. For all items, individuals responded on a 6-point Likert scale (1=strongly disagree, 6=strongly agree) and a mean score is created for each subscale separately. Higher mean scores on each subscale indicate greater endorsement of that mindset. The BMI-C is similar to the adult version^23,26^ but with the language simplified to make the scale suitable for children and adolescents. See Table S2, Supplemental Digital Content 2, http://links.lww.com/CJP/B213 for items from the BMI-C.

#### Pain Acceptance: Pain Willingness and Activity Engagement

Pain acceptance was measured using the 8-item short form Chronic Pain Acceptance Questionnaire for Adolescents (CPAQ-A8) subscales.^31^ The CPAQ-A8 includes 2 subscales: pain willingness (4-items; α=0.74), and activity engagement (4-items; α=0.82). Pain willingness refers to the willingness to let pain be present without effort to control pain; per the scoring procedure, these items are reverse-scored.^32^ Activity engagement refers to perseverance with personally valued actions in the presence of pain.^32^ Responses are provided on a 5-point Likert scale across items (0=never true, 4=always true). Items from each subscale are summed, where higher scores indicate greater activity engagement and pain willingness.

#### Mental Health Symptoms

##### Anxiety and Depression

The Patient Reported Outcome Measurement Information System (PROMIS) pediatric anxiety and depression measures assessed depressive and anxious symptoms over the past 7 days on a 5-point Likert scale (1=never, 5=almost always). PROMIS pediatric measures were administered through computer adaptive testing (CAT) using item response theory (IRT). CAT administers different questions to participants using questions from item banks based on answers to prior questions. A minimum of 5 questions are given to create a T-score per measure (M=50, SD=10). As more questions are answered, the error decreases and confidence in the T-score increases. Questions will continue to be administered until either the standard error drops below a prespecific level (4.0 for the pediatric measures) or the participant has answered the maximum number of items. PROMIS pediatric measures have been validated in children aged 8 to 17 years.^33^ Participant T-scores from these measures were obtained from the Peds-CHOIR registry; therefore, Cronbach alphas could not be computed.

#### Basic Functioning

The Functional Disability Inventory (FDI) was utilized as a measure of self-reported basic functioning. The FDI contains 15 items that assess children’s perceived difficulty in performing everyday activities in school, home, physical, and social contexts (α=0.89).^34^ Items are rated on a 5-point Likert scale ranging from 0 (no trouble) to 4 (impossible). Items are summed to create a total score; higher scores indicate poorer basic functioning.

#### Additional Pain-Related Risk and Resilience Factors

##### Pain Catastrophising

The Pain Catastrophising Scale for Children (PCS-C) contains 13 items that assess rumination, magnification, and helplessness about pain.^35^ Individuals respond on a 5-point Likert scale (0=not at all, 4=extremely) and higher scores indicate greater pain catastrophising. Cronbach alpha was 0.91 for the total score.

##### Pain-Related Fear and Avoidance

The 10-item short form Fear of Pain Questionnaire Child Version (FOPQC-SF) assesses pain-related fear and avoidance.^36^ The FOPQC-SF contains 2 subscales: fear of pain (4-items; α=0.70) and avoidance of activities (6-items; α=0.71). Responses are provided on a 5-point Likert scale (0=strongly disagree, 4=strongly agree). Items for each subscale are summed, where higher scores reflect higher levels of pain-related fear and avoidance. A total score of fear avoidance was also created by summing items from both subscales. Cronbach alpha was 0.77 for the total score.

##### Pain-Related Self-Efficacy

Self-efficacy for functioning despite chronic pain was assessed using the child version of the Pain Self-Efficacy Scale (PSES-C).^37^ The PSES-C contains 7-items that are measured on a 5-point Likert scale (1=very sure, 5=very unsure). Items are summed to create a total score where lower scores indicate higher pain self-efficacy. Total scores for this measure were obtained from the Peds-CHOIR registry; therefore, Cronbach alpha could not be computed.

### Statistical Analyses

Data were analysed using SPSS version 28,^38^ with significance levels set at *P*<0.05 and 2-tailed. Descriptive statistics were used to summarize demographic variables and pain characteristics. The distribution of the body mindset subscales were presented visually using R version 4.1.3.^39^ Pain duration was calculated as the number of days in pain from the initial pain date reported by parents to the survey start date. In 7 cases, parents reported their child’s pain duration as <3 months. Patients with pain lasting under 3 months may still attend the Stanford pain clinic if their pain had persisted beyond the typical healing timeframe and if acute aetiologies had been effectively excluded. Sensitivity analyses were conducted to examine correlations between study variables and pain duration, both including and excluding the 7 cases with reported pain durations of <3 months. Relationships between study variables with pain duration, including and excluding the 7 cases did not substantially differ, thus analyses proceeded including these 7 cases (see Table S3, Supplemental Digital Content 3, http://links.lww.com/CJP/B214).

Pearson correlation analyses and independent-sample *t* tests were conducted to examine univariate relationships among demographic factors, pain characteristics, pain acceptance, body mindsets, basic functioning, mental health symptoms, and additional pain-related risk and resilience factors. A post-hoc power calculation was performed using G*Power version 3.1.^40^ Observed correlations between body mindsets and study variables ranged from *r*=0.20 and *r*=0.40 (see Results section). With a sample size of 102 participants and a significance level of *P*<0.05 (2-tailed), the study was adequately powered to detect correlations of *r>*0.30 (88% power) and *r>*0.40 (99%).

Hierarchical linear regression analyses were conducted to examine the associations between body mindsets and each subscale of pain acceptance—namely, activity engagement and pain willingness—while controlling for demographic and pain characteristics, and basic functioning. In step 1, age and sex were added to the models. In step 2, pain characteristics, including pain frequency, average pain intensity, pain location (localized vs. diffuse) and pain duration were added. In step 3, basic functioning was added. Finally, in steps 4a, 4b, and 4c, each body mindset—Body is Capable, Body is Responsive, and Body is an Adversary—were added individually. In step 5, all 3 body mindsets were added simultaneously to assess the total variance in pain acceptance explained by body mindsets beyond basic functioning, demographics and pain characteristics, and to identify which specific mindset was most relevant to each aspect of pain acceptance. All variables were added using the enter method.

## RESULTS

### Sample Demographic and Pain Characteristics

The sample included 102 adolescents with chronic pain, with a mean age of 13.76 years (SD=2.53). Of the sample, 72.3% female, and 49.5% self-identified as Caucasian/white. Most reported diffuse pain (65.3%). In the past month, 70.3% of participants reported pain daily, with an average pain intensity of 5.52 out of 10 (SD=1.87). Pain duration varied widely, ranging from 37 to 5479 days (∼15 y), with a median duration of 858 days (∼2.5 y). Full medical and demographic data are presented in Table 1. Missing data across all items was minimal (3.05%). Analyses included participants who completed at least 80% of the items on each scale, with no imputation performed for missing values.

**TABLE 1:** Demographic and Medical Characteristics of the Sample

Variable	Category	M (SD)	Range	*%*
Age	—	13.76 (2.53)	8–17	—
Sex	Male	—	—	26.7
	Female	—	—	72.3
Race	White	—	—	49.5
	Asian	—	—	1
	Native Hawaiian or other Pacific Islander	—	—	1
	Declines to state	—	—	7.9
	Unknown	—	—	20.8
	Other			10.9
	Missing			8.9
Pain location	Head	—	—	46.5
	Face or Jaw	—	—	7.9
	Shoulder or Neck	—	—	36.6
	Chest	—	—	12.9
	Arm or Hand	—	—	26.7
	Abdomen or Stomach	—	—	41.6
	Back	—	—	30.7
	Leg or Foot	—	—	44.6
	Other	—	—	10.9
Binary pain location groupings	Localized	—	—	34.7
	Diffuse	—	—	65.3
Pain frequency	Daily	4.36 (1.21)	0-5	70.3
	Five to six days per week	—	—	11.9
	Two to three days per week	—	—	8.9
	Once per week	—	—	3
	About one to three times per month	—	—	4
	No pain in the last month	—	—	2
Average pain intensity	—	5.52 (1.87)	0-10	—
Pain duration*	—	1350.54 (1338.41)	37-5479	—

* Pain duration is reported in days.

*Note. Pain duration is reported in days.

### Variations in Body Mindsets

**FIGURE 1:**
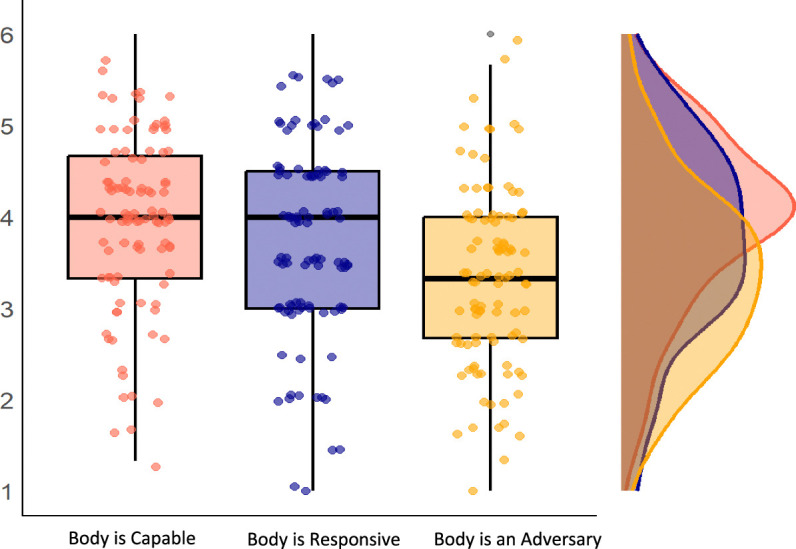
Variations in body mindsets for adolescents with chronic pain.

As illustrated in Figure 1, scores on each body mindset subscale were widely distributed, reflecting substantial individual variability in the mindsets that adolescents with chronic pain hold about their bodies. The median endorsement for the Body is Capable mindset fell within the “somewhat agree” range (Mdn=4.00, IQR=1.33). In contrast, the median score for the Body is an Adversary (Mdn=3.33, IQR=1.33) and Body is Responsive (Mdn=3.75, IQR=1.50) mindsets ranged from “somewhat agree” to “somewhat disagree.” To quantify endorsement levels for each mindset, scores on each subscale were categorized as either agreement or disagreement. Specifically, participants with scores ranging from 1 to 3 were considered to disagree with endorsing the mindset, while those with scores >3 up to 6 were considered to agree with endorsing the mindset. Overall, 81.2% (n=82) of participants endorsed the mindset that my Body is Capable, 56.4% (n=57) endorsed the mindset that my Body is an Adversary, and 67.3% (n=68) endorsed the mindset that my Body is Responsive.

As shown in Table 2, older adolescents with chronic pain were more likely to endorse the mindset that my Body is an Adversary. Age was not significantly associated with the endorsement of the Body is Responsive or Body is Capable mindsets. Males (M=4.11, SD=1.08) were more likely to endorse the mindset that my Body is Responsive compared with females (M=3.57, SD=1.08). The Body is Responsive and Body is Capable mindsets were positively and moderately associated with each other, while the Body is an Adversary mindset was not significantly associated with either of the other mindsets, indicating that the Body is an Adversary mindset may be conceptually separate from the other, more helpful body mindsets (Table 3).

**TABLE 2:** Demographic Correlates of Body Mindsets, Pain, Pain Acceptance, Pain-Related Risk and Resilience Factors, and Mood-Related Factors.

	Sex (*t*)	Age (*r*)
Body mindsets		
Body is capable	−0.665	0.114
	0.508	0.257
Body is responsive	2.210*	0.153
	0.029	0.126
Body is an adversary	−1.459	0.287*
	0.148	0.004
Pain characteristics		
Pain frequency	−1.817	0.093
	0.072	0.300
Average pain intensity	0.650	−0.073
	0.517	0.418
Pain duration	−0.626	0.049
	0.533	0.632
Pain-related risk and resilience factors		
Fear of pain	0.591	−0.089
	0.556	0.389
Avoidance	−0.133	0.220*
	0.894	0.031
Fear avoidance	0.618	0.100
	0.538	0.332
Pain catastrophising	−0.504	0.017
	0.615	0.866
Self-efficacy	1.627	0.094
	0.108	0.400
Pain acceptance		
Pain willingness	0.605	0.000
	0.547	0.999
Activity engagement	−1.437	0.044
	0.154	0.663
Total pain acceptance	−0.421	0.038
	0.675	0.707
Mental health symptoms		
Depression	−0.155	0.097
	0.877	0.313
Anxiety	−0.468	0.006
	0.641	0.958
Basic functioning		
Functional disability inventory	1.182	0.072
	0.240	0.476

* *P*<0.05.

For Sex effects, positive *t* values represent boys >girls.

The top line for each variable represents the *t* or *r* value, the second line represents the *P*-value.

**TABLE 3:** Associations of Body Mindsets with Pain, Pain Acceptance, Pain-Related Risk and Resilience Factors, and Mental Health Symptoms

	1.	2.	3.	4.	5.	6.	7.	8.	9.	10.	11.	12.	13.	14.	15.
Body mindsets															
Body is capable	1														
Body is responsive	0.489** <0.001	1													
Body is an adversary	−0.084 0.404	−0.103 0.305	1												
Pain characteristics															
Pain frequency	−0.028 0.778	−0.090 0.371	0.000 1.00	1											
Average pain intensity	−0.169 0.091	−0.211* 0.034	−0.123 0.220	0.141 0.159	1										
Pain duration	0.033 0.748	0.073 0.477	0.079 0.447	0.032 0.752	−0.093 0.366	1									
Pain acceptance															
Pain willingness	0.091 0.369	0.065 0.523	−0.283** 0.005	−0.184 0.068	0.023 0.818	0.026 0.806	1								
Activity engagement	0.435** <0.001	0.272** 0.006	−0.231* 0.021	0.072 0.478	−0.216* 0.032	0.072 0.487	0.263** 0.009	1							
Pain-related risk and resilience factors															
Fear of pain	−0.095 0.357	−0.080 0.436	0.287** 0.005	0.015 0.881	0.170 0.098	−0.034 0.752	−0.353** <0.001	−0.229* 0.026	1						
Avoidance	−0.170 0.098	−0.050 0.629	0.287** 0.005	0.152 0.140	0.108 0.295	0.068 0.521	−0.438** <0.001	−0.271** 0.008	0.416** <0.001	1					
Fear avoidance	−0.163 0.114	−0.075 0.467	0.367*** <0.001	0.109 0.201	0.160 0.119	0.028 0.790	−0.483** <0.001	−0.302** 0.003	0.797** <0.001	0.881** <0.001	1				
Pain catastrophising	−0.322** 0.001	−0.285** 0.004	0.276** 0.006	0.024 0.815	0.322** 0.001	−0.055 0.597	−0.205* 0.044	−0.238* 0.019	0.555** <0.001	0.262** 0.010	0.463** <0.001	1			
Self-efficacy	−0.197 0.077	−0.138 0.215	0.089 0.426	−0.045 0.685	0.342** 0.002	−0.138 0.227	−0.223* 0.047	−0.467* <0.001	0.317** 0.004	0.505** <0.001	0.506** <0.001	0.317** 0.004	1		
Mental health symptoms															
Anxiety	−0.151 0.163	−0.151 0.163	0.224* 0.037	0.115 0.287	0.158 0.143	−0.077 0.487	−0.245* 0.024	0.185 0.096	0.652** <0.001	0.188 0.084	0.472** <0.001	0.443** <0.001	0.185 0.096	1	
Depression	−0.116 0.284	−0.089 0.410	0.149 0.170	0.071 0.513	0.175 0.105	0.068 0.541	−0.030 0.784	0.114 0.307	0.388** <0.001	0.126 0.250	0.290** 0.007	0.328** 0.002	0.114 0.307	0.648** <0.001	1
Basic functioning															
Functional disability inventory	−0.095 0.345	−0.063 0.530	0.139 0.165	0.303** 0.002	0.411** <0.001	0.029 0.778	−0.214* 0.034	−0.264** 0.008	0.191 0.062	0.420** <>001	0.379** <0.001	0.126 0.213	0.554** <0.001	0.297** 0.005	0.298** 0.005

Top line for each variable represents *r* value, second line represents *P*-value.

* *P*<0.05.

** *P*<0.01.

*** *P*<0.001.

### Correlations Between Body Mindsets, Pain Characteristics, and Basic Functioning

As shown in Table 3, adolescents who endorsed the mindset that my Body is Responsive reported lower average pain intensity, whereas endorsement of the other mindsets were not significantly associated with average pain intensity. Endorsement of body mindsets were not significantly associated with individual differences in pain frequency, pain duration, or pain location (Body is an Adversary; *t*=−0.113, *P*=0.911, Body is Capable; *t*=1.12, *P*=0.267, Body is Responsive; *t*=0.907, *P*=.367). None of the body mindsets were significantly associated with basic functioning.

### Body Mindsets, Pain Willingness, and Activity Engagement: Correlations and Regression Analyses

As shown in Table 3, adolescents with chronic pain who more strongly endorsed the Body is Capable mindset reported greater activity engagement. Adolescents who more strongly endorsed the Body is Responsive mindset also reported greater activity engagement. In contrast, adolescents who more strongly endorsed the Body is an Adversary mindset reported both lower pain willingness and lower activity engagement.

In a hierarchical linear regression model with pain willingness as the outcome variable, the Body is an Adversary mindset was significantly associated with pain willingness after controlling for demographics, pain characteristics, and basic functioning. The other mindsets did not contribute unique variance to the outcome of pain willingness. Overall, all 3 body mindsets contributed 6.6% of the variance in pain willingness and all variables in the model contributed 8.1% variance in pain willingness (Table 4).

**TABLE 4:** Regression Analyses of Body Mindsets Predicting Pain Willingness

	Step 1	Step 2	Step 3	Step 4a	Step 4b	Step 4c	Step 5
Demographic factors							
Age	0.03 (-0.24- 0.30)	0.06 (−0.20 to 36)	0.08 (−0.17 to 1.24)	0.07 (−0.19 to 0.37)	0.09 (−0.16 to 0.39)	0.15 (−0.09 to 0.46)	0.16 (−0.07 to 0.49)
Sex	−0.08 (−2.10 to 0.98)	−0.05 (−1.97 to 1.31)	−0.07 (−.2.15 to 1.11)	−0.08 (−2.18 to 1.09)	−0.10 (−2.34 to 0.99)	−0.01 (−1.70 to 1.56)	−0.07 (−2.20 to 1.19)
Pain-related factors							
Average pain intensity		0.02 (−0.37 to 0.44)	0.10 (−0.24 to 0.61)	0.11 (−0.22 to 0.64)	−08 (−0.30 to 0.58)	0.10 (−0.24 to 0.59)	0.07 (−0.31 to 0.54)
Pain duration		−0.02 (0.00- 0.00)	−0.002 (0.00- 0.00)	−0.001 (0.00- 0.00)	−0.004 (0.00- 0.00)	0.02 (0.00-0.00)	0.01 (0.00- 0.00)
Pain frequency		−0.22 (−1.22 to 0.01)	−0.16 (−1.07 to 0.19)	−0.16 (−1.07 to 0.18)	−0.16 (−1.07 to 0.18)	−0.18 (−1.10 to 0.12)	−0.18 (−1.11 to 0.10)
Pain location		−0.04 (−1.75 to 1.24)	0.01 (−0.153 to 1.44)	0.004 (−1.47 to 1.53)	−0.02 (−1.64 to 1.37)	0.01 (−1.41 to 1.48)	−0.003 (−1.48 to 1.44)
Basic functioning							
Functional disability inventory			−0.23 (−0.14 to 0.004)	−0.23 (−0.14 to 0.004)	−0.23 (−0.14 to 0.005)	−0.18 (−0.13 to 0.02)	−0.17 (−0.13 to 0.02)
Body mindsets							
Body is capable				0.09 (−0.45 to 1.05)			0.16 (−0.30 to 1.38)
Body is responsive					−0.09 (−0.91 to 0.38)		−0.21 (−1.34 to 0.12)
Body is adversary						−0.27* (−1.60 to −0.15)	−0.27* (−1.64 to −0.18)
*Adj R* ^2^	−0.016	−0.014	0.015	0.011	0.081	0.069	0.081
*F* statistic	0.287	0.790	1.20	1.12	1.13	1.83	1.79

First value represents the standardized beta estimate, followed by the 95% CI.

* *P*<0.05.

In a hierarchical linear regression model with activity engagement as the outcome variable, all 3 body mindsets were significantly associated with activity engagement when entered individually. The Body is an Adversary mindset was associated with lower activity engagement, while the Body is Capable and Body is Responsive mindsets were associated with higher activity engagement. When all 3 mindsets were entered into the same model (step 4), both the Body is Capable and Body is an Adversary mindsets remained significantly associated with activity engagement. Together, the 3 body mindsets contributed 21.8% of the variance in activity engagement and all variables in the model contributed 26.8% variance in activity engagement (Table 5).

**TABLE 5:** Regression Analyses of Body Mindsets Predicting Activity Engagement

	Step 1	Step 2	Step 3	Step 4a	Step 4b	Step 4c	Step 5
Demographic factors							
Age	0.02 (−0.23 to 0.27)	−0.04 (−0.30 to 0.22)	−0.02 (−0.27 to 0.24)	−0.05 (−0.29 to 0.16)	−0.05 (−0.31 to 0.18)	0.06 (−0.18 to 0.33)	0.003 (−0.23 to 0.24)
Sex	0.15 (−0.412 to 2.46)	0.10 (−0.83 to 2.23)	0.07 (−1.01 to 2.00)	0.05 (−0.99 to 1.71)	0.15 (−0.48 to 2.51)	0.14 (−0.52 to 2.46)	0.13 (−0.55 to 2.29)
Pain-related factors							
Average pain intensity		−0.18 (−68 to 0.06)	−0.09 (−0.54 to 0.25)	−0.02 (−0.39 to 0.32)	−0.01 (−0.41 to 0.37)	−0.09 (−0.54 to 0.22)	−0.02 (−0.34 to 0.32)
Pain duration		0.08 (0.00- 0.00)	0.10 (0.00- 0.00)	0.11 (0.00- 0.00)	0.11 (0.00- 0.00)	0.12 (0.00- 0.00)	0.13 (0.00- 0.00)
Pain frequency		0.11 (−0.29 to 0.85)	0.18 (−0.12 to 10.04)	0.17 (−0.07 to 0.97)	0.18 (−0.08 to 1.03)	0.16 (−0.14 to 0.98)	0.16 (−0.10 to 0.92)
Pain location		−0.03 (−1.56 to 1.21)	0.01 (−1.31 to 1.44)	0.07 (−0.82 to 1.67)	0.05 (−1.02 to 1.65)	0.02 (−1.17 to 1.50)	0.08 (−0.72 to 1.72)
Basic functioning							
Functional disability inventory			−0.27* (−0.15 to −0.10)	−0.27* (−0.14 to −0.02)	−0.27* (−0.14 to −0.01)	−0.21 (−0.13 to 0.01)	−0.23* (−0.13 to −0.01)
Body mindsets							
Body is capable				0.43*** (0.80- 2.05)			0.37*** (0.52- 1.94)
Body is responsive					0.29* (0.23- 1.40)		0.06 (−0.44 to 0.78)
Body is adversary						−0.29** (−1.58 to −0.25)	−0.23* (−1.35 to −0.11)
*Adj R* ^2^	0.001	0.003	0.050	0.231	0.120	0.117	0.268
*F* statistic	1.02	1.05	1.68	4.42***	2.55*	2.51*	4.33***

First value represents the standardized beta estimate, followed by the 95% CI.

* *P*<0.05.

** *P*<0.01.

*** *P*<0.001.

### Exploratory Correlations Between Body Mindsets and Additional Pain-Related Risk, Resilience, and Mental Health Factors

As shown in Table 3, we observed additional correlations between body mindsets and pain-related risk, resilience, and mental health factors, which may inform future research directions. Adolescents who endorsed the mindset that my Body is an Adversary reported greater pain-related fear, avoidance, and pain catastrophizing, while those more strongly endorsing the Body is Capable and my Body is Responsive mindsets reported less pain catastrophizing. There were no significant associations between body mindsets and pain self-efficacy. Regarding mental health symptoms, adolescents who endorsed the Body is an Adversary mindset reported higher anxiety, but no significant associations were found with depressive symptoms. The Body is Capable and Body is Responsive mindsets were not significantly associated with symptoms of anxiety or depression.

## DISCUSSION

Our results provide initial evidence that adolescents with chronic pain hold diverse mindsets about the function and capabilities of their bodies. These mindsets do not simply appear to be reflections of pain severity, mental health status, or basic functioning—indeed, mindsets were not significantly correlated with these factors. Instead, body mindsets were associated with adolescents’ level of pain acceptance. Consistent with hypotheses, adolescents who viewed their bodies as not working properly or letting them down (as an “adversary”) reported significantly lower willingness to experience pain. In contrast, those who viewed their bodies as responsive and capable of managing pain showed greater engagement in valued activities despite pain. In hierarchical linear regression analyses, body mindsets accounted for unique variance in pain acceptance beyond basic demographics, pain characteristics, and everyday functioning.

The presented data are cross-sectional, and they represent only a preliminary investigation of the role of body mindsets in the motivational context of living with chronic pain. In experimental research outside the context of pain, several studies have shown that mindsets influence behavior by shaping motivational processes. For example, perceiving stress as “enhancing” rather than “debilitating” has increased individuals’ willingness to engage in challenging situations, such as receiving feedback after giving a speech, by highlighting the potential for growth.^21^ Similarly, adopting the mindset that healthy eating and exercise are “appealing” can enhance motivation to engage in health behaviors, including increasing physical activity and maintaining a healthy diet.^41^ It remains to be determined whether body mindsets may similarly guide motivation in adolescents with chronic pain, for example, to pursue meaningful activities and influence how they use their bodies while experiencing pain. Although causality cannot be established in the current study, the mindsets adolescents hold about their bodies may guide their engagement in activities despite pain or motivate efforts to manage it. These behaviors, in turn, could reinforce specific body mindsets. Longitudinal research investigating the dynamic interplay between mindsets and motivational engagement in the context of pain is warranted.

Emerging evidence from experimental studies suggests that body mindsets, like other mindsets, are modifiable. For instance, “Wise Intervention” strategies, which introduce helpful mindsets during periods of uncertainty, have positively impacted well-being, health, and behavior.^25^ A brief digital intervention targeting body and illness mindsets improved coping, quality of life, and symptom distress in cancer patients, with changes in the mindset that the Body is Capable partially mediating improvements in quality of life during cancer treatment.^26^ These cross-sectional data provide support for conducting similar experimental studies to determine the causal role of body mindsets in shaping motivational processes and pain acceptance in youth with chronic pain. In particular, intervention strategies that seek to lessen the mindset my Body is an Adversary and to instil the mindset my Body is Capable are particularly warranted. Of note, while mindsets accounted for a relatively larger proportion of variance in activity engagement, they contributed relatively limited variance to pain willingness. Within the psychological flexibility model, the behavioral response of willingness is not dependent on specific patterns of cognition.^18^ Nonetheless, certain mindsets may promote willingness to a degree, as seen here. Using Wise Intervention strategies alongside other approaches that are well-established to foster pain acceptance, for example, acceptance and commitment therapy, could therefore be useful.^18^

We observed several additional findings of interest. First, median scores on the Body is an Adversary subscale of the BMI-C were slightly higher among adolescents with chronic pain (Mdn=3.33, SD=0.98) compared with those reported in previous research with survivors of childhood cancer (Mdn=2.75, SD=1.19; Dowling et al., 2023). In contrast, median scores on the Body is Capable (Mdn=4.00, SD=1.00) and Body is Responsive (Mdn=3.75, SD=1.10) subscales were slightly lower in adolescents with chronic pain than in childhood cancer survivors (Capable: Mdn=4.50, SD=2.10; Responsive: Mdn=4.00, SD=1.26). These findings suggest that adolescents with chronic pain may marginally endorse the Body is an Adversary mindset more strongly, and the Body is Capable and Body is Responsive mindsets less strongly than those who have survived childhood cancer. Childhood cancer survivors may experience a sense of having overcome a major health challenge, potentially promoting a stronger belief in the body’s resilience and capabilities compared with adolescents with chronic pain that might present as more of a persistent and ambiguous health challenge. Although SDs for all subscales were slightly lower in the chronic pain sample, the differences were small, suggesting comparable levels of variability across groups. Visual inspection of boxplots (Fig. 1 in the current study and Figure 2 in Dowling et al., 2023) that illustrate the distribution of scores further supports this, indicating similar broad variability in endorsement of body mindsets in both samples. Second, body mindsets varied by demographic factors. Older adolescents were more likely to endorse the mindset that their Body is an Adversary, aligning with research showing that health mindsets change with age and experience.^42–44^ Moreover, males were more likely to endorse the mindset that their Body is responsive compared with females, which aligns with broader trends in body image literature wherein females typically report more negative perceptions of body functionality.^45–47^ In addition, adolescents who endorsed the Body is Responsive mindset reported experiencing less intense pain, consistent with research showing that body mindsets relate to pain severity in other health populations.^22^ When experiencing long-term pain, believing the body can eventually “heal” and recover from pain may help mitigate the negative experience of ongoing pain. Notably, there were no significant associations between body mindsets and pain location or duration, suggesting that body mindsets are not solely influenced by duration or spread of pain. Interestingly, while body mindsets were associated with adolescents’ engagement with valued life activities despite pain (ie, activity engagement), they were not associated with basic functioning (eg, difficulty walking to the bathroom or walking up stairs). In other words, youth who held the mindset that their Body is Capable were unlikely to report greater activity engagement simply because they were objectively functioning better. This provides preliminary evidence that mindsets more relevant for understanding pain-related behaviors within a motivational context—given the complex interplay between basic functioning and activity engagement in chronic pain populations, considering mindsets provides an interesting avenue for future research.

Pain catastrophising, fear of pain, and avoidance are well-established factors that contribute to pain persistence and disability and were assessed during the initial clinic evaluation for our sample.^48,49^ Since mindsets are proposed to broadly influence affect, attention, behavior, and physiology,^25^ we explored correlations of body mindsets with these additional pain-related risk and resilience factors to yield insights for future research. Our findings show that adolescents who endorsed the mindsets that their Body is Capable or their Body is Responsive were less likely to engage in pain catastrophising. In contrast, those who endorsed the mindset that their Body is an Adversary reported higher levels of catastrophising, fear of pain, and avoidance behaviors. Viewing the body as an untrustworthy adversary may increase concern about pain leading to disengagement, while seeing the body as capable may reduce pain threat appraisal and rumination. Moreover, adolescents who endorsed the mindset that their Body is an Adversary reported greater symptoms of anxiety, possibly due to uncertainty about their body’s reliability. However, body mindsets were not significantly associated with symptoms of depression, suggesting they are not merely reflections of low mood. Interestingly, body mindsets also did not significantly correlate with pain self-efficacy, indicating that mindsets may be distinct from a general sense of confidence in managing pain.

Body mindsets align closely with the concept of “Core Beliefs” as defined by extensive bodies of research.^50–54^ These studies suggest that behavior arises from complex interactions between how individuals interpret both themselves and their environment. It is not only the objective situation that determines behavior, but also the subjective meaning a person assigns to the context and circumstance, especially in ambiguous contexts where multiple interpretations are possible. Because people can interpret the same situation or aspect of themselves in different ways, their responses often vary accordingly. This body of research helps explain individual differences in behavior and well-being by showing that interpretations are often shaped by core beliefs. As such, it supports interventions that intentionally target and shift these interpretive processes to promote helpful outcomes. Body mindsets could thus also be referred to as “Core Body Beliefs”; whether the term “mindset” holds more appeal in therapeutic interventions remains to be established. Nonetheless, the current study thus contributes to the growing evidence that beliefs about the body and pain play a role in chronic pain outcomes.^55–58^ Clinical guidelines recommend addressing unhelpful beliefs as the first line of treatment for chronic musculoskeletal pain.^59^ However, most research has focused on beliefs about the causes of chronic pain, for example, whether pain is attributed to unresolved tissue damage.^60^ Other research has measured “illness perceptions” regarding the longevity, curability, and controllability of pain (eg, “my pain will last forever” or “nothing I do will affect my pain”).^61,62^ While these beliefs may predict pain self-management, they may not be the most effective therapeutic targets. Clinicians, especially those with their own biomedical beliefs about pain, may struggle to counteract these beliefs in patients.^59^ Even if patients change their beliefs about the cause or curability of their pain, these new beliefs can be challenged by persisting pain or conflicting messages from health care providers. In line with the Crum and Clifton (under review) definition, Core Beliefs may be particularly useful therapeutic targets as they often do not represent an objective “truth.” In the case of Core Beliefs about stress, one can find ample evidence that stress is both debilitating and enhancing.^20^ Similarly, for Core Beliefs about the world, there is ample evidence that the world is both a dangerous and safe place.^52^ Similarly, individuals can find evidence that their body is capable of handling pain (my Body is Capable), can heal (my Body is Responsive), or is vulnerable and failing them (my Body is an Adversary). In fact, one can find evidence to support each of these mindsets throughout life, both at times of sickness and health. Given that these core beliefs about the body do not represent an objective truth, they may be less easily refuted by new evidence, making them promising targets for interventions. Whether body mindsets are malleable and impactful, particularly in the context of chronic pain, warrants investigation.

This study has several limitations, which suggest directions for future research. As noted above, the cross-sectional design prevents conclusions about causality. Body mindsets may influence adolescents’ motivation to engage in valued activities or prioritize pain control, or it could be that engaging in valued life activities shapes helpful body mindsets. A third, unstudied factor could also underlie the observed association between body mindsets and activity engagement. Longitudinal and experimental research is needed to determine the strength of directional effects between beliefs, motivation, and behaviors in this context. Relatedly, while a strength of this study is the inclusion of a clinical sample, we were unable to collect follow-up data to examine whether body mindsets predicted treatment outcomes. Future studies should investigate whether the mindsets youth hold when first attending a pain clinic predict treatment or response. Whether body mindsets predict the transition from acute to chronic pain would also be of interest. Regarding associational limitations, the study was underpowered to detect associations with small effect sizes. In addition, we did not assess precipitating events or history of diagnoses, which could influence body mindsets and warrant further investigation. Fourth, pubertal-related changes and development, which were not assessed in this study, may influence body mindsets in adolescent chronic pain. Future research should consider pubertal stage as a relevant factor when exploring body mindsets in adolescent populations and how this is relevant for associations with pain acceptance. Fifth, the Cronbach alpha for the Body is an Adversary subscale was lower than in other populations. This likely reflects the coherence of the conceptualization of the Body is an Adversary mindset. The belief that the body is to blame for experiencing pain may represent a distinct construct from the belief that the body is broken for experiencing pain, which may be particularly salient in the context of chronic pain compared with other health conditions. This lower internal consistency might have undermined associations between the Body is an Adversary mindsets and other study variables. Future research should further explore this potential distinction and examine whether relationships with relevant variables differ depending on the specific belief endorsed. Future mixed-methods research could further explore body mindsets in individuals with chronic pain, both clarifying existing body mindsets as measured by the BMI and identifying additional mindsets that may serve as effective treatment targets. For example, mindsets framing the body as safe (versus dangerous) or communicative (versus uncommunicative) could be relevant and may be addressed in current pain treatments.^63^ Finally, the sample was recruited from a single center and mainly comprised females. Examining body mindsets across diverse populations, including various age groups, cultural backgrounds, pain conditions, and clinical settings, is needed to understand the generalizability of these findings, particularly for underrepresented groups.

Despite limitations, this study introduces a novel focus on body mindsets in the context of chronic pain. Our findings suggest that adolescents with chronic pain hold diverse mindsets about the functionality of their bodies, and that the degree to which youth hold these mindsets is associated with their level of pain acceptance. In hierarchical linear regression analyses, body mindsets contributed unique variance to pain acceptance beyond demographic factors, pain severity, and basic functioning. These results point towards future research to explore whether body mindsets may serve as valuable targets in multidisciplinary pain management, in particular to enhance activity engagement and thereby to improve overall outcomes for youth with chronic pain.

## Supplementary Material
