# Supplementary material for: Pain Acceptance in Adolescent Chronic Pain: Do Body Mindsets Play a Role?
Source: Clin J Pain. 2025 Jul 1;41(9):e1307. doi: 10.1097/AJP.0000000000001307 (PMC12341747; doi:10.1097/AJP.0000000000001307)
Supplement: Supplementary file 1 [file ajp-41-e1307-s001.docx]

**TABLE S1**. Sensitivity Analyses Comparing Differences in Variables Between those who Completed the BMI-C and those who did not.

|  |  | Those who did not complete the BMI-C (*M*, SD) | Those who completed the BMI-C (*M*, SD) | *t* | *p* |
| --- | --- | --- | --- | --- | --- |
| Demographic Factor | Age | 14.36 (2.36) | 13.76 (2.53) | 1.07 | .29 |
| Pain Characteristics | Pain Frequency | 4.38 (1.02) | 4.36 (1.21) | .11 | .91 |
|  | Average Pain Intensity | 6.35 (1.94) | 5.52 (1.87) | 1.98 | .05 |
| Pain-Related Risk & Resilience Factors | Fear of Pain | 7.36 (3.93) | 7.54 (3.38) | -.19 | .85 |
|  | Avoidance | 14.64 (6.02) | 14.23 (4.31) | 1.07 | .28 |
|  | Fear Avoidance | 23.00 (8.82) | 21.78 (6.50) | .63 | .53 |
|  | Pain Catastrophising | 28.92 (9.94) | 26.70 (10.60) | .74 | .46 |
|  | Self-Efficacy | 23.33 (4.09) | 20.10 (5.80) | 2.55 | .01 |
| Mental Health Symptoms | Depression | 57.29 (12.07) | 54.91 (8.81) | 1.07 | .28 |
|  | Anxiety | 53.42 (9.30) | 52.44 (10.04) | .43 | .67 |

**Note.** Statistics for Basic Functioning, Pain Acceptance, Pain Willingness, and Activity Engagement could not be completed due to there being one individual in the group that did not complete the BMI-C.
