# Supplementary material for: Pain Acceptance in Adolescent Chronic Pain: Do Body Mindsets Play a Role?
Source: Clin J Pain. 2025 Jul 1;41(9):e1307. doi: 10.1097/AJP.0000000000001307 (PMC12341747; doi:10.1097/AJP.0000000000001307)
Supplement: Supplementary file 2 [file ajp-41-e1307-s002.docx]

**TABLE S2.** Items from the Body Mindset Inventory-Child Version Oriented Around the Context of Pain.

| **My Body is Capable** |
| --- |
| My body can handle pain |
| My body is designed to deal with pain |
| My body is capable |
| **My Body is Responsive** |
| My body can heal itself |
| Most of the time, my body can heal on its own |
| **My Body is an Adversary** |
| My body is to blame for the pain I feel |
| Having pain means that my body is letting me down |
| Having pain means that my body isn’t working right |

**Note.** All items are rated on a 6-point Likert scale (1= strongly disagree, 6= strongly agree).
