# Supplementary material for: Pain Acceptance in Adolescent Chronic Pain: Do Body Mindsets Play a Role?
Source: Clin J Pain. 2025 Jul 1;41(9):e1307. doi: 10.1097/AJP.0000000000001307 (PMC12341747; doi:10.1097/AJP.0000000000001307)
Supplement: Supplementary file 3 [file ajp-41-e1307-s003.docx]

**TABLE S3.** Sensitivity Analyses Comparing Correlations in the Sample with and without Participants Reporting Pain Duration Exceeding 3 Months.

|  |  | Pain Duration (including data >3 months) | Pain Duration (excluding data >3 months) |
| --- | --- | --- | --- |
| Body Mindsets | Body is Capable | .029  .778 | .040  .711 |
|  | Body is Responsive | .072  .481 | .098  .368 |
|  | Body is an Adversary | -.035  .733 | -.042  .693 |
| Pain Characteristics | Pain Frequency | .032  .752 | .077  .470 |
|  | Average Pain Intensity | -.093  .366 | -.101  .341 |
| Pain-Related Risk & Resilience Factors | Fear of Pain | -.012  .253 | -.123  .263 |
|  | Avoidance | -.072  .494 | -.093  .395 |
|  | Fear Avoidance | -.111  .291 | -.128  .245 |
|  | Pain Catastrophising | -.233*  .023 | -.241*  .024 |
|  | Self-Efficacy | -.087  .448 | -.060  .609 |
| Pain Acceptance | Pain Willingness | -.123  .232 | .098  .362 |
|  | Activity Engagement | .241*  .019 | .215*  .045 |
| Mental Health Symptoms | Depression | .017  .879 | .023  .844 |
|  | Anxiety | -.031  .777 | -.032  .779 |
| Basic Functioning | Functional Disability Index | .029  .787 | .042  .695 |

Data on the top line represents the *r* value, data below represents the *p* value.
